# Moving Toward Patient-Tailored Treatment in ALS and FTD: The Potential of Genomic Assessment as a Tool for Biological Discovery and Trial Recruitment

**DOI:** 10.3389/fnins.2021.639078

**Published:** 2021-03-01

**Authors:** Iris J. Broce, Patricia A. Castruita, Jennifer S. Yokoyama

**Affiliations:** ^1^Memory and Aging Center, Department of Neurology, Weill Institute for Neurosciences, University of California, San Francisco, San Francisco, CA, United States; ^2^Department of Family Medicine and Public Health, University of California, San Diego, San Diego, CA, United States; ^3^Department of Radiology and Biomedical Imaging, University of California, San Francisco, San Francisco, CA, United States

**Keywords:** clinical trials, genomics, precision medicine, amyotrophic lateral sclerosis, frontotemporal dementia

## Abstract

Amyotrophic lateral sclerosis (ALS) and frontotemporal dementia (FTD) are two devastating and intertwined neurodegenerative diseases. Historically, ALS and FTD were considered distinct disorders given differences in presenting clinical symptoms, disease duration, and predicted risk of developing each disease. However, research over recent years has highlighted the considerable clinical, pathological, and genetic overlap of ALS and FTD, and these two syndromes are now thought to represent different manifestations of the same neuropathological disease spectrum. In this review, we discuss the need to shift our focus from studying ALS and FTD in isolation to identifying the biological mechanisms that drive these diseases—both common and distinct—to improve treatment discovery and therapeutic development success. We also emphasize the importance of genomic data to facilitate a “precision medicine” approach for treating ALS and FTD.

## Introduction

Amyotrophic lateral sclerosis (ALS) and frontotemporal dementia (FTD) are two devastating and intertwined neurodegenerative diseases. ALS is a progressive and fatal motor neuron disease (MND), leading to muscle atrophy, paralysis, and eventual death from respiratory failure within 3–5 years from symptom onset (Rowland and Shneider, 2001). FTD is characterized by changes in social behavior and/or language abilities at disease onset due to neurodegeneration of the frontal and temporal lobes, leading to death within 3–12 years from symptom onset (Mitchell et al., 2015; Kansal et al., 2016; Ahmed et al., 2020). Historically, ALS and FTD were considered distinct disorders given differences in presenting clinical symptoms, disease duration, and predicted risk of developing each disease. However, research over recent years has highlighted the considerable clinical, imaging, pathological, and genetic overlap of ALS and FTD, and these two syndromes are now thought to represent different manifestations of the same neuropathological disease spectrum.

Amyotrophic lateral sclerosis and FTD represent opposite ends of the same disease continuum, defined by underlying TDP-43 neuropathology (Geser et al., 2010). Indeed, the terms ALS and FTD remain useful for clinical diagnostic and prognostic purposes. Patients clinically diagnosed with pure forms of FTD have a prolonged survival compared to patients diagnosed with pure ALS and patients diagnosed with combined ALS-FTD (Hodges et al., 2003; Govaarts et al., 2016; Kansal et al., 2016; Xu et al., 2017). We speculate that distinct molecular etiologies—driven in part through genetic differences—result in distinct clinical manifestations of disease from a common neuropathological entity. Although there are currently no disease-modifying drugs that halt or reverse the progression of either ALS or FTD, leveraging both the unique and shared genetic contributions to ALS and FTD may facilitate therapeutic discovery. In this review, we discuss the need to shift our focus from studying ALS and FTD in isolation to identifying the biological mechanisms that drive these diseases—both common and distinct—to improve treatment discovery and therapeutic development success. We also emphasize the importance of genomic data to facilitate a “precision medicine” approach for treating ALS and FTD.

## Clinical Phenotypes of FTD and ALS

Frontotemporal dementia is an umbrella term and represents one of the most common forms of dementia diagnosed in people younger than 65 years old. The global prevalence and incidence of FTD is uncertain, with estimates among people in the United States aged 45–64 between 15–22 per 100,000 and 2.7–4.3 per 100,000 person-years, respectively (Onyike and Diehl-Schmid, 2013; Knopman and Roberts, 2011; Logroscino et al., 2019; Turcano et al., 2020). Incidence of FTD increases with age (Rosso et al., 2003; Logroscino et al., 2019) and is roughly equal between males compared to females, though studies report greater risk in males (Onyike and Diehl-Schmid, 2013; Turcano et al., 2020). Depending on the signs and symptoms, FTD patients are classified into one of three different clinical syndromes: behavioral variant FTD (bvFTD) or one of two forms of primary progressive aphasias (PPA), including non-fluent variant PPA (nfvPPA) and semantic variant PPA (svPPA). People with FTD may also experience movement symptoms, such as bradykinesia, dystonia, rigidity, and apraxia, with 12.5% of patients with bvFTD meeting clinical criteria for either corticobasal syndrome (CBS) or progressive supranuclear palsy syndrome (PSPS) (Ljubenkov and Miller, 2016). About 15% of people with bvFTD, 11% of patients with nfvPPA, and 19% of patients with svPPA may also eventually develop motor symptoms consistent with ALS (Rascovsky et al., 2011; Vinceti et al., 2019).

Amyotrophic lateral sclerosis is the most common form of adult-onset MND. It is classically characterized by progressive degeneration of *both* upper motor neurons (UMN) of the motor cortex and lower motor neurons (LMN) of the brainstem and spinal cord at disease onset. Although motor neuron damage is predominating in ALS, other neurons in the fronto-executive circuits, temporal and parietal cortical regions, basal ganglia, and dorsal root ganglia are also involved in some patients (Bede et al., 2013; Westeneng et al., 2016; Cykowski et al., 2017; Omer et al., 2017; Nakamura-Shindo et al., 2020; Riancho et al., 2020). ALS is considered a rare “orphan” disease. The global prevalence and incidence of ALS is 4.42 (95% CI 3.92–4.96) per 100,000 and 1.59 (95% CI 1.39–1.81) per 100,000 person-years, respectively (Xu et al., 2020). ALS prevalence and incidence increases with age until about the age of 70–79 and is higher in males compared to females (Xu et al., 2020). Because ALS shows extensive phenotypic heterogeneity in disease presentation, redefining ALS as a group of clinical syndromes is gaining favor (Kim et al., 2009; de Vries et al., 2019; Shefner et al., 2020). For example, according to the current consensus, primary lateral sclerosis (PLS), which results from UMN loss exclusively, and progressive muscular atrophy (PMA), which results from LMN loss exclusively, are distinct clinical phenotypes of MND relying on a continuum with ALS. This poses a delicate diagnostic problem since some patients initially diagnosed with PLS or PMA eventually develop LMN and UMN loss, respectively, and meet clinical criteria for ALS later on (Gordon et al., 2006; Kim et al., 2009). Several factors, including survival, distinguish PLS or PMA from ALS, with shorter survival in ALS patients (Turner et al., 2020). Further, about 50% of ALS patients develop cognitive and behavioral impairment, with 13% meeting diagnostic criteria for bvFTD (Ljubenkov and Miller, 2016). Recently, one study showed that cognitive and behavioral impairment, including FTD, are also common in PLS and PMA (de Vries et al., 2019). Together, these findings provide valuable insight into ALS’s clinical heterogeneity and overlapping clinical features between MNDs and FTD.

The FTD and ALS clinical syndromes described above represent the manifestations of underlying neuropathology that results in the dysfunction and death of neurons in specific neuroanatomical regions. That is: individuals with bvFTD manifest with dysexecutive and behavioral symptoms because neurons within specific regions of the brain underlying executive function and social behavior (e.g., anterior cingulate, frontoinsular, striatum, and amygdala regions) are impacted (Perry et al., 2017); individuals with svPPA manifest semantic loss because neurons within anterior temporal lobe related to semantic knowledge are impacted (Gorno-Tempini et al., 2011). Neuroimaging studies additionally support the notion of ALS and FTD as a continuum showing that motor cortex and anterior cingulate as well as their underlying white matter tracts are impacted in ALS patients, while widespread frontal, anterior cingulate, insular, and temporal lobes are impacted in ALS-FTD and bvFTD patients (Lillo et al., 2012; Crespi et al., 2018; Trojsi et al., 2018; Seeley, 2019; Vinceti et al., 2019). Furthermore, while most forms of ALS are due to pathological inclusions of TDP-43 (with some exceptions, noted in the next section), about half of all bvFTD (Neumann et al., 2006; Perry et al., 2017), most svPPA (Grossman, 2010; Josephs et al., 2011; Borghesani et al., 2020), and a portion of nfvPPA cases are due to TDP-43 (Adams-Carr et al., 2020). The other half of bvFTD, a small minority to few svPPA, over half of nfvPPA, CBS, and PSPS result from underlying tau pathology (Josephs et al., 2011).

## Shared Genetic Risk Highlights Molecular Overlap of FTD and ALS

Since the clinical phenotypes of FTD and ALS can be heterogeneous, it may seem that these syndromes have different underlying biological mechanisms. However, clinical, genetic, and neuropathological overlap between these diseases is well-established (Lomen-Hoerth et al., 2002; DeJesus-Hernandez et al., 2011; Renton et al., 2011; Weishaupt et al., 2016; Abramzon et al., 2020). About 30 disease-causing mutations have been repeatedly associated with ALS (for a review see, Abramzon et al., 2020). Fewer genes have been associated with FTD. The most common disease-causing mutations occur in chromosome 9 open reading frame 72 (*C9ORF72*) and progranulin (*GRN*), which both result in TDP-43 neuropathology, and microtubule-associated protein tau (*MAPT*), resulting in tau neuropathology (Olney et al., 2017). The most common causes of either ALS, FTD, or combined ALS and FTD (ALS-FTD), even within the same families, are pathogenic hexanucleotide repeat expansions in *C9ORF72*. Nearly 25–40% of all familial ALS and FTD cases carry this mutation and 5–7% of sporadic cases—cases without an established family history of neurodegenerative disease—also screen positive for *C9ORF72* pathogenic expansions (DeJesus-Hernandez et al., 2011; Renton et al., 2011). These estimates are based on European ancestry; *C9ORF72* mutations are relatively rare in people of Asian ancestry, and other ancestral populations remain understudied (Majounie et al., 2012). Beyond *C9ORF72*, mutations in several other genes have been associated with both ALS and FTD, including *TARDBP, SQSTM1, VCP, FUS, TBK1, CHCHD10*, and *UBQLN2* (Abramzon et al., 2020). The pathological hallmark of ALS and FTD patients harboring these particular mutations, with the exception of *FUS*, is the presence of ubiquitinated protein deposits primarily composed of TDP-43. How mutations in the same genes cause different clinical syndromes despite similar neuropathology remains unknown and may relate to differences in mutation localization on downstream processes or modifying genetic and/or environmental factors. Nevertheless, shared genetic contributions to FTD and ALS suggest that there are, at least in part, common molecular mechanisms driving disease pathology. Novel disease-modifying treatments are underway for ALS and FTD patients that target specific molecular subtypes. Since half of FTD patients show TDP-43 proteinopathy and the other half shows tau proteinopathy, it will be increasingly important to use genetic information to accurately predict the underlying pathology.

Characterizing genotype-phenotype interactions in both ALS and FTD have diagnostic and prognostic value and can help in selecting patients for clinical trials. A known genotype/phenotype association provides information about how certain genes or genetic variants result in specific features of ALS or FTD, including symptoms at onset, severity of motor/cognitive impairment, rate of disease progression, and survival. These genotype-phenotype interactions for ALS and FTD have been recently reviewed in prior reports (Van Mossevelde et al., 2018; Connolly et al., 2020). ALS patients with specific *TARDBP* mutations show differences in survival; patients with G298S mutations have shorter survival (27 months) compared to patients with A315T and M337V mutations (100 months) (Regal et al., 2006). Patients with A4V *SOD1* mutations have shorter survival (less than 12 months) compared to patients with other mutations, such as D90A or G93C (5–10 years) (Cudkowicz et al., 1997; Regal et al., 2006; Corcia et al., 2012). Patients with *TARDBP* and *SOD1* mutations have earlier onset (53.4, 50.1 years, respectively) compared to sporadic cases (61.9 years) (Corcia et al., 2012). For patients with mutations in *FUS*, age of onset depends on the location of the mutation and type of mutations (missense, nonsense, and deletion) (Waibel et al., 2013). Different mutations have been associated with different symptoms at onset – higher proportion of patients with mutations in *SOD1*, *hnRNAP1*, and *TUBA4A* manifest with limb onset (>80%), compared to patients with mutations in other genes: *VCP* (50%), *NEK1* (50%), and *TBK1* (50%), *C9orf72* (33%), *UBQLN2* (40%) (Connolly et al., 2020). Also, the prevalence of cognitive impairment and FTD in ALS varies in cases with different mutations: *C9orf72*, *SQSTM1*, *TBK1, TARDBP* (36, 67, 43, and 12%, respectively) (Connolly et al., 2020). Furthermore, people with mutations in different genes show differing prevalence of FTD subtypes (Van Mossevelde et al., 2018). FTD patients who carry mutations in *C9ORF72, GRN, TBK1*, and *VCP* are all associated with TDP-43 pathology. However, patients with *C9ORF72* repeat expansions with bvFTD often present with psychotic symptoms, whereas patients with FTD who carry a *GRN* mutation often present with apathetic behavior and language impairment. Patients with FTD who carry *TBK1* mutations often present with MND and language and behavioral impairment but no psychotic symptoms, whereas patients with FTD who have a *VCP* mutation may present with or without myopathy or Paget disease of the bone and show apathy, anomia, and/or psychotic symptoms. Knowing that patients with FTD carrying mutations in *C9ORF72* or *VCP* are likely to show psychotic symptoms is valuable because clinicians can anticipate complications of these symptoms, with the potential of managing the condition more effectively. In sum, known genotype-phenotype relationships provide information about how genes may work in causing certain features (e.g., age of onset, survival, and clinical features such as concomitant FTD and psychosis or Paget disease). This information helps clinicians in estimating prognosis, provides insight into molecular mechanisms of motor neuron cell death, and can also provide a framework to test new therapies.

In familial forms of ALS and FTD, the underlying neuropathology is predictable. However, in sporadic disease, there is more variability, particularly in FTD. Given that sporadic ALS and FTD may have distinct molecular etiology of disease and future therapeutics will likely target a specific protein or biological pathway (e.g., inflammation), it will be critical to have tools in place that can effectively distinguish between these *in vivo*. PET ligand-based imaging has begun to try to address this question through tau imaging in FTD (Dani et al., 2016; Tsai et al., 2019) and translocator protein (TSPO) PET imaging, a neuroinflammation biomarker, in ALS (Van Weehaeghe et al., 2020). However, numerous technical challenges remain to be addressed. In addition to lack of specificity, PET imaging represents an expensive procedure dependent upon specific infrastructure to create the radioligand. Additionally, TSPO PET studies require genotyping of the rs6971 polymorphism to determine TSPO binding affinity since approximately 5–10% of individuals may have a lower specific signal (Owen et al., 2012; Van Weehaeghe et al., 2020). For this reason, molecular-based biomarkers such as genetic profiles represent an appealing means of identifying underlying neurodegenerative disease pathology in a relatively inexpensive, non-invasive way. Indeed, individuals with genetic forms of FTD and their unaffected family members are currently being recruited for longitudinal study, setting the stage for clinical trials in large families with predictable disease trajectories (Tsai and Boxer, 2016; Rosen et al., 2020). Although current clinical trials in ALS only recruit symptomatic cases despite evidence that ALS has a long presymptomatic phase (Eisen et al., 2014), clinical trials aimed at better understanding the clinical and biological changes that occur in individuals with genetic forms of ALS and are asymptomatic are underway^1^. Taken together, there is a critical need for identifying novel molecular mechanisms driving sporadic ALS and FTD pathogenesis and a critical need for developing novel mechanism-based biomarkers that can assist in patient selection for molecular-based clinical trials. Also, studies of familial disease provide an opportunity to test early-stage or even presymptomatic disease given the established underlying disease etiology.

## Technological Advancement Drives Genetic Discovery

To facilitate identification of new treatment approaches and preventative measures, FTD and ALS genetics research seeks to elucidate the underlying mechanisms of disease; these biological discoveries can then be probed as potential targets for disease modification. The human genome contains about 3 billion base pairs of DNA, which reside within the nucleus of every cell of the body. Perhaps unsurprisingly, the genomes of any two unrelated individuals are more similar than different since genomes define our species. At the same time, no two individuals–even identical twins–are genetically identical (Vadgama et al., 2019; Homfray, 2020). Strong efforts from various international groups, including 1000 Genomes Project and the Genome Aggregation Database (gnomAD), have curated comprehensive catalogs of human genetic variation by genotyping and sequencing hundreds of thousands of individuals from diverse populations (Genomes Project et al., 2015; Koch, 2020). These large databases serve as a global reference for human genetic variation. A genetic variant is a term used to refer to differences in a DNA sequence (e.g., C and T) at a specific location between two genomes (e.g., patient genome compared to human reference genome). The genome of any two unrelated individuals shows differences in roughly 0.5% of their genome, which equals approximately 15 million genetic variants (Jackson et al., 2018). In this context, researchers aim to identify genetic variants that are enriched in patients with ALS and FTD versus unaffected controls.

Next-generation sequencing (NGS) technologies and high-throughput genotyping platforms have revolutionized the ability to detect genetic variation in humans. Genotyping-based approaches use microarray technology, which is well-established, highly accurate, and less expensive than NGS. Clinicians are very familiar with these tests, helping maintain their place as a critical diagnostic tool. However, microarray-based approaches are limited in number of genetic variants they can probe and are only able to survey “known” variants by nature of their design. Thus, microarray technology is better suited for profiling well-established single nucleotide polymorphisms (SNPs) or established disease-causing genetic variants but not suitable for discovering novel or extremely rare genetic variants that have not been observed in previously generated datasets.

Next-generation sequencing refers to high-throughput technology that determines the sequence of nucleotides across an entire surveyed region, including genome (whole-genome sequencing; WGS), exons within all known genes (whole-exome sequencing; WES), or pre-selected coding or non-coding regions that target only a portion of the genome (target panel). Unlike genotyping, there is no requirement for *a priori* knowledge of the genetic variants of interest. We can identify known genetic variants and novel genetic variants that may be unique to each patient. NGS technology also enables us to capture multiple types of genetic variants, including SNVs, indels, structural variants, common and rare variants in the population: common variants [minor allele frequency (MAF) ≥ 5%], low-frequency variants (0.5% ≤ MAF < 5%), rare variants (MAF < 0.5%), and *de novo* mutations. While WGS in particular seems promising in providing an all-in-one solution for detecting multiple types of variants, there are several drawbacks. It is expensive relative to other sequencing and microarray technologies on a per-sample basis, though given its flexibility in detecting multiple variants, the per-patient cost is cheaper (van Nimwegen et al., 2016; Schwarze et al., 2020). WGS also requires robust bioinformatics capabilities for variant annotation, classification, interpretation and enormous amounts of space and server capabilities for processing, storing, and backing up the data in a reasonable timeframe. Finally, variant detection relies on DNA quality, sequencing coverage, fidelity of the reference assembly, and the number and population background of samples being assessed. Given these technical complexities and constraints in infrastructure and resources, WGS has been slower to enter the clinical space. Identifying ways to overcome these challenges will be critical to translating these genomic advances into patient care.

## Genomic Contributions to Familial and Sporadic ALS and FTD

An important question is, how much does genetics matter for predicting ALS and FTD risk in non-familial forms of disease? The term heritability is a statistical concept used to describe the proportion of phenotypic variation that can be explained by genetic variation. Heritability estimates are important and widely used for determining how well we can predict a trait from genetics. Heritability estimates range from 0 to 1, with a score of 1 indicating that genetics explains all the variance in the trait and a score of zero indicating that it explains none. The heritability estimates of ALS based on pedigree and twin-studies is moderate to high, ranging between 0.40–0.60 and 0.38–0.78, respectively (Al-Chalabi et al., 2010). The heritability estimates of ALS based on genome-wide genotyping SNP data is 0.12–0.21 which is low-to-moderate (Fogh et al., 2014; Keller et al., 2014; McLaughlin et al., 2015). Interestingly, all the genetic signal from the largest sporadic ALS GWAS to date, which is based on 36,052 people (12,577 cases and 23,475 controls), comes from chromosome 9 (van Rheenen et al., 2016; Holland et al., 2021). Therefore, ALS GWAS, based on SNP data, may not capture rare variants or low-frequency variants pertinent to ALS. Additionally, since the current ALS GWAS is underpowered, the extent to which increases in sample size can help identify novel SNPs in other regions remains an important question. Collectively, these findings suggest that genetic factors do play an important role in predicting ALS risk. Increases in sample sizes may help explain additional phenotypic variance from SNP data beyond the signal from *C9ORF72*. The genetic contribution to ALS may be underestimated based on the way heritability is calculated (e.g., using pedigree information, twin data, or case-control data and with genome-wide SNP data, WES, or WGS) and the variant information generated through different genotyping/sequencing technologies (e.g., low-frequency, rare variants, structural variants, or *de novo* variants). Taken together, these studies provide justification for continued efforts toward data collection in additional ALS patients to enhance the field’s ability to identify novel genetic variants influencing disease risk. Indeed, WGS data collection efforts in ALS patients would enable capturing the full spectrum of genetic variation—including rare variants—associated with disease risk and may provide novel insights into additional genetic contributions to ALS.

The heritability estimates in FTD based on pedigree data range between 0.26 and 0.31 (Rohrer et al., 2009; Wood et al., 2013; Greaves and Rohrer, 2019). These estimates vary across the different clinical subtypes, with bvFTD showing highest heritability at 0.58, compared to svPPA at 0.22, nfvPPA 0.30, and 0.10–0.40 in FTD-ALS (Goldman et al., 2005; Rohrer et al., 2009). Genetic variants captured by exome array data (predominantly low frequency) explains 53% of the total phenotypic variance of sporadic FTD (excluding FTD-MND patients) (Chen et al., 2015). However, whether common variants explain a significant portion of phenotypic variance in FTD is also difficult to say. The small sample size and wide range of clinical and neuropathological variability pose important challenges in estimating FTD heritability. Despite these limitations, as shown in the original FTD GWAS paper, the per-variant effect size (odds ratio ≈ 1.3) found in the major histocompatibility complex (MHC) area on chromosome 6 is quite strong (Ferrari et al., 2014). This indicates the need for complex haplotype analysis, which could be addressed through access to sequencing data. WGS and WES in sporadic disease has only begun to emerge and several mutations and CNVs have been identified in 11% of sporadic cases (Blauwendraat et al., 2018). Taken together, the genetic architecture of FTD is highly complex. Low-frequency, rare variants and CNVs may likely explain more genetic variation in sporadic FTD compared to common variants. Further, better understanding the complex haplotype driving risk associations in the MHC region may improve both prediction and inference of the genetic contribution to FTD risk.

## Genetic Influences on Disease Beyond Risk

In addition to overall disease risk, genetic information can inform our understanding of how individual differences in people’s genes affects their response to drugs (pharmacogenetics). Pharmacogenetics is important for improving targeted treatment and minimizing adverse drug reactions. For example, recent ALS research has shown that genetic variants can modify the effects of drugs on patient outcomes. Lithium carbonate, which is proposed to boost autophagy and remove misfolded proteins that accumulate in motor neurons during the course of disease, was found not to benefit people with ALS in a past clinical trial. However, a recent *post hoc* meta-analysis across three clinical trials found that the effect of lithium was dependent on patient genotype (van Eijk et al., 2017). Individuals with a particular genotype at a SNP in *UNC13A* (C/C genotype; rs12608932) showed better 12-month survival compared to non-carriers and the control group. Similarly, in separate *post hoc* analysis of two other randomized clinical trials, the same authors evaluated whether the effect of creatine monohydrate and valproic acid also depended on the genotype of patients. They found a dose-dependent pharmacogenetic interaction between creatine and the A allele of a SNP (i.e., rs616147) in *MOBP*, suggesting a qualitative interaction in a recessive model (van Eijk et al., 2020). These studies highlight the importance of incorporating genetic information into clinical trials to identify patient subgroups that are most likely to benefit from a particular treatment, either through direct or indirect effects of genetic variation on drug metabolism, disease biology and/or downstream processes influenced by the intervention. Results from conducting pharmacogenetic *post hoc* analyses may serve as a powerful method for refining the inclusion criteria for subsequent trials and minimizing the sample size required to detect a therapeutic effect.

We may also be able to improve treatment discovery and success rates by leveraging the observation that ALS and FTD—particularly in their familial forms—represent a disease spectrum. Rather than studying diseases in isolation or restricting inclusion criteria to patients with a single clinical syndrome, it may be more useful to modify inclusion criteria for clinical trials and recruit patients based on a common genetic etiology and/or molecular phenotype of disease, regardless of showing different clinical syndromes (e.g., PLS, ALS, and FTD).

**FIGURE 1:**
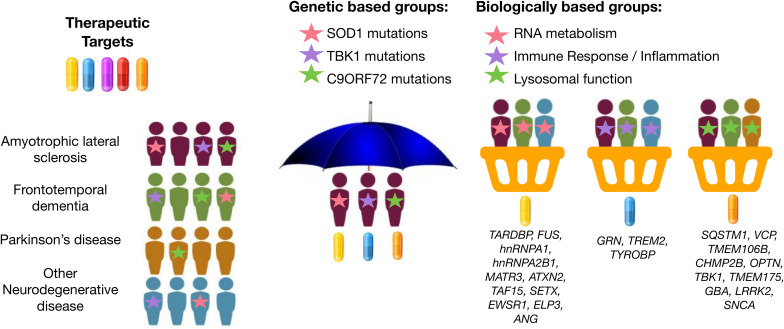
Graphical representation of umbrella and basket trials. Trials have shifted from enrolling patients from a single clinical syndrome and genetic mutation for a selective therapeutic target (umbrella trial) to enrolling patients across different clinical syndromes for treatment using a therapeutic target most relevant for a given molecular or biological pathway implicated by underlying genetic risk (basket trial). Genes were mapped to biologically based groups based on two recent review papers (Wallings et al., 2019; Liscic et al., 2020).

Basket design clinical trials, which have been widely used in oncology research, are a newer trial design that offers this type of flexibility. Basket trials allow investigation of therapeutic efficacy of a candidate therapeutic simultaneously in multiple clinical syndromes that result from the same genetic or molecular aberration (Figure 1). In this framework, patients can be assessed for treatment response using individualized endpoint measures that are most relevant to their diagnosis. This method was recently attempted in neurodegenerative disease. For example, Tsai et al. (2020) assessed the safety, tolerability, and pharmacodynamics of the microtubule stabilizer TPI-287 in Alzheimer’s disease (AD) and in the 4-repeat tauopathies (4RT) PSPS and CBS. They found that patients with AD tolerated TPI-287 less than those with 4RT as a result of the presence of anaphylactoid reactions. Similar to this tau-based study, basket trial approaches could be applied to ALS and FTD by grouping patients with similar genetic forms of disease. If we can use genetic information to identify specific molecular profiles unique to each patient’s particular form of disease, we may be able to leverage genetic information to drive a “precision medicine” strategy for disease treatment, even at the clinical trial phase.

Similarly, genetic information may be useful in identifying the most relevant therapeutic target(s) based on each patient’s specific etiology or molecular profile of disease. Several new potential drugs are currently being tested in Phase 1 to Phase 3 clinical trials for ALS and FTD: therapeutic approaches to neuroinflammation (e.g., masitinib and *Cannabis sativa*), autophagy and protein quality control (e.g., arimoclomol and NCT03491462), apoptosis (e.g., tauroursodeoxycholic acid; TUDCA), mitochondria and endoplasmic reticulum function (e.g., AMX0035), among others. The various genes associated with ALS and FTD encode proteins associated with different classes of cellular processes (Yousefian-Jazi et al., 2020). For example, *GRN, TREM2*, and *TYROBP* are implicated in immune response and neuroinflammation (Jay et al., 2017; Bright et al., 2019; Rostalski et al., 2019); *SOD1, CHCHD10*, and *C19ORF12* are implicated in mitochondrial and oxidative stress (Deschauer et al., 2012; Tan et al., 2014; Anderson et al., 2019); and *EWSR1, FUS, SETX, TAF15*, and *TARDBP* are implicated in DNA damage and repair (Higelin et al., 2016; Hill et al., 2016). Rather than randomly assigning patients to the placebo and treatment group in a drug-mediated clinical trial, it may prove useful to instead profile patients for deleterious variants in genes associated with one of these common disease-associated biological pathways. Then, enroll patients into basket trials for treatment using a therapeutic candidate most relevant for the given pathway implicated by underlying genetic risk (i.e., target neuroinflammation in individuals with risk profiles consistent with immune dysregulation) (Figure 1).

Taken together, collecting genetic information in ALS and FTD patients is important for investigating the pharmacogenetic interactions in clinical trials, improving recruitment accuracy for clinical trials, and may accelerate drug discovery.

## Leveraging Bioinformatics Innovation for Gene Discovery in ALS and FTD

There are several analytic techniques that exist for gene discovery and that provide insights into the disease mechanisms of ALS and FTD. Many research groups have exploited pleiotropic methods for gene discovery. Genetic pleiotropy refers to the phenomenon of one gene influencing two or more phenotypes. Beyond *C9ORF72*, studies have identified CAG repeat expansion in the ataxin-2 gene (*ATXN2*) as a genetic cause of spinocerebellar ataxia type 2 (SCA2) and ALS (Elden et al., 2010; Lee et al., 2011; Li et al., 2016; Sproviero et al., 2017). Independent GWAS have identified pleiotropy between FTD and other diseases, such as AD (Ferrari et al., 2017). Using novel pleiotropic methods based on the Bayesian conditional FDR (cFDR) approach (Andreassen et al., 2013; Broce et al., 2019; Frei et al., 2019), we have discovered pleiotropic loci between FTD and ALS beyond *C9ORF72*, as well as pleiotropy between FTD and, CBD and PSP (Yokoyama et al., 2017), and between FTD and other less clearly related traits, such as immune-mediated diseases (Broce et al., 2018). The discovery of pleiotropic genes and biological pathways shared across diseases may be leveraged for repurposing drugs already approved for disorders beyond ALS and FTD and may facilitate basket trials that recruit patients with different diseases that may respond to similar treatments.

In addition to novel analytical methods, machine-learning based approaches are also being leveraged for gene discovery (Grollemund et al., 2019). Recently, one study used phenotypic and biological information from publicly available databases and applied a knowledge graph edge prediction algorithm to predict novel ALS-associated genes (Grollemund et al., 2019; Bean et al., 2020). Across various approaches, they identified over 500 predicted genes linked to ALS. The predicted new candidate genes were linked to a number of biological processes previously associated with ALS, including angiogenesis, lipid metabolism, mitochondria activity, protein kinase activity, superoxide metabolism, vesicle-trafficking, and neurotransmitter regulation. These candidate genes now require validation in large, independent cohorts that include rare and common genetics variants; these data are currently lacking. Thus, as more and more individuals are sequenced, larger patient datasets will be generated that can facilitate validation of these findings and discovery of additional genes, which will both be critical next steps required before this information can be applied to a broader spectrum of patients and used to identify at-risk individuals.

It is important to note that the general consensus is that ALS and FTD have a complex genetic architecture. While a single gene might be associated with increased risk for disease in one patient (monogenic), a few genes might be associated with increased risk in another patient (oligogenic), and many genes may be associated in a third patient (polygenic). Future studies may benefit from implementing polygenic risk scoring approaches incorporating newly identified risk loci. These scores create single, continuous measures composed of the sum of small to moderate contributions from tens to thousands of single genetic variants. In the future, polygenic risk scores could be used to create a personalized genetic diagnostic tool that stratifies people into clear trajectories for disease risk and outcome, and, ultimately, could even inform therapeutic decision-making.

## Personalized Genomics to Facilitate Clinical Care

Global collaboration between medical centers, academic research institutions, non-profit, and for-profit organizations is necessary to dissect the genetic heterogeneity in ALS and FTD and accelerate discovery of treatments for all patients. No universal database that stores genetic information and other biomarkers for all ALS and FTD patients currently exists. Having a uniform database or registry of both ALS and FTD patients would enable identification of all patients with specific genetic mutations and facilitate analyses that leverage the genetic overlap between diseases. This, in turn, would accelerate recruitment for gene-focused clinical trials and research to identify novel disease-modifying therapies. Currently, different organizations (e.g., AnswerALS^2^, ProjectMinE^3^, TargetALS^4^, ALLFTD^5^, dbGAP^6^, and GENFI^7^) are leading separate efforts in ALS or FTD. While select groups have plans to ‘‘unite’’ data across studies, open collaboration across geographic regions and granting bodies requires orchestration across a number of challenging fronts. Issues ranging from data normalization and warehousing infrastructure to regulatory agencies and federal guidelines create enormous barriers that will need to be overcome to accelerate global genomic efforts. Also, ALS and FTD are rare diseases, and the percent of people with particular genetic mutations is even rarer. For example, based on three national United States databases (2010--2011), the Center for Disease Control and Prevention reports that about 12,000--15,000 people currently had ALS^8^. Of these, roughly 1,200–1,500 people would have familial form of ALS (10% of all ALS cases) and 10,800–13,500 would have sporadic form of ALS (90% of all ALS cases). Of these, about 120–150 people (10% of all familial ALS cases) would have *SOD1* mutations and about 216–540 people (2–4% of all sporadic ALS cases) would also be expected to harbor *SOD1* mutations. Therefore, approximately 400–700 ALS patients in the United States would have *SOD1* mutations. These approximations are likely underestimated since records on ALS have not been systematically collected throughout the country. In light of this example, it is not surprising that it takes years to recruit the required number of patients for clinical trials. Furthermore, FTD patients are most often diagnosed in memory clinics, while ALS patients are seen in movement disorder clinics; despite the biological overlap and frequent comorbidity of these disorders, both patient groups would benefit from the expertise of these two siloed specialist fields. A unified database would facilitate recruitment for gene-focused clinical trials across both disorders.

The current guidelines for genetic testing in patients with ALS, FTD, or ALS-FTD are conservative. A multi-step process for genetic testing in ALS, FTD, or ALS-FTD patients has been recently proposed (Roggenbuck and Fong, 2020). All patients with ALS or FTD are recommended genetic screening for the *C9ORF72* repeat expansion. However, only patients with a family history of either condition or early onset of symptoms are recommended for comprehensive multigene panel testing as a second step. Most ALS/FTD panels include roughly 30 genes. A family history of dementia or motor-neuron disease is not always present for particular mutations (e.g., *MAPT* mutations). Incomplete penetrance of genetic variants that do not always confer disease or have variable age of onset, inaccurate or incomplete family history information (e.g., adoption), misdiagnosis, early death, and other factors also reduce accuracy of records of family history (Roggenbuck and Fong, 2020). Although a family history is useful for genetic counseling, there is no clear biological distinction between familial and sporadic forms of ALS or FTD. Therefore, such stipulations limit a patient’s ability to get genetic testing, which in turn limits their access to cutting-edge treatments. Further, while certain mutations (e.g., *C9ORF72*) may be more common in one population (European vs. Asian), ancestry should not limit a patient’s ability to access genetic testing. Patients of diverse populations should be able to access information from genetic testing so they, too, can enroll into drug-target clinical trials, which do not exclude patients on the basis of ancestry (e.g., SOD1 AMX035; NCT03127514). Indeed, safety, tolerability, and pharmacodynamics of the therapeutic targets should be assessed in diverse populations during Phase 1 clinical trials. In sum, there are several limitations in the way that the current genetic testing guidelines are designed and recommendations for genetic testing are being made, and these may result in limited access to treatment trials, particularly for individuals from diverse backgrounds.

In this context, it would benefit all patients to have equitable access to WGS and accompanying genetic counseling such that patients can be provided access to relevant genetic information as it becomes clinically actionable. Since no cure for ALS or FTD currently exists, genetic testing for these diseases has been of minimal priority for insurance companies. However, as more genes are discovered that perspective is changing. Some research institutions, such as University of California, San Francisco are now offering genome sequencing paid by research to all of their patients to spearhead their precision medicine health program^9^. Further, we may improve patients’ care as well as gene discovery if patients who consent to WGS/WES were permitted to consent to enrolling in a data registry for the purpose of collecting evidence of a genotype–phenotype relationship (Holtzman, 2013). Strong efforts are being made to revise the ethical guidelines of reporting genetic findings to ALS/FTD patients (Leighton et al., 2019; Shoesmith et al., 2020). Close collaboration between physicians, large ALS centers, non-profit and for-profit institutions, ethicists, genetic counselors, and data scientists will be critical for carrying out these tasks that, while complicated, also have the potential to facilitate a tailored approach to clinical care and treatment in ALS and FTD.

## Concluding Remarks

Taken together, ALS and FTD are now thought to represent different manifestations of the same disease spectrum. The field is starting to move toward basket design clinical trials that allow investigation of drug-mediated treatments simultaneously in different clinical syndromes that involve the same genetic or molecular aberration. The genetic architecture of ALS and FTD is complex; different types of genetic variants (e.g., common, rare, low-frequency, and CNV) in different genes cause either ALS, FTD or ALS-FTD in different people. For this reason, WGS is appropriate technology to facilitate identification of disease-causing genetic variants in a single patient and discovery of novel disease-causing genetic variants in large-scale studies. WGS may be particularly valuable for characterizing diverse and understudied populations, where novel variants may be associated with disease and/or where differences in genomic background may modify the clinical presentation of mutations in a given gene. We know that the heritability of ALS and FTD is high, which provides rationale for continuing to expand genomic research in these conditions. Novel approaches for gene discovery, such as machine learning and leveraging pleiotropy, are rapidly growing in ALS and FTD research. In turn, new clinical trials driven by these novel findings will likely be soon underway. In addition to informing therapeutic development, genetic information will be valuable for assessing pharmacogenetic interactions in clinical trials and may ultimately be leveraged to stratify patients to optimize therapeutic efficacy. In this way, collection of genetic data is critical for enhancing our understanding of safety, tolerability, and individual difference in response to therapies. Because ALS and FTD are rare diseases, global collaboration between physicians, large neurodegenerative disease centers, non-profit and for-profit institutions, and other key domain experts will be critical for identifying effective treatments that will stop or slow the progression of disease in clinically and demographically diverse ALS and FTD patients.

## Author Contributions

IB wrote the initial draft of the manuscript. PC and JY edited the manuscript. All authors contributed to the article and approved the submitted version.

## Conflict of Interest

The authors declare that the research was conducted in the absence of any commercial or financial relationships that could be construed as a potential conflict of interest.

## Footnotes

1 https://www.neals.org/als-trials/1330

2 https://www.answerals.org

3 https://www.projectmine.com

4 https://www.targetals.org

5 https://www.allftd.org

6 https://www.ncbi.nlm.nih.gov/gap/

7 https://www.genfi.org

8 https://www.cdc.gov/als/

9 https://www.3dhealthstudy.org
